# Fragmented QRS complexes on a 12-lead ECG as a marker of non-coronary artery disease related myocardial disease by gadolinium delayed enhancement cardiac magnetic resonance imaging

**DOI:** 10.1186/1532-429X-11-S1-P58

**Published:** 2009-01-28

**Authors:** Mohamed Homsi, Lamaan Alsayed, Anas Safadi, Mithilesh K Das, Jo Mahenthiran

**Affiliations:** Indiana University School of Medicine, Krannert Institute of Cardiology, Indianapolis, IN USA

**Keywords:** Amyloidosis, Cardiac Magnetic Resonance, Cardiac Magnetic Resonance Imaging, Hemochromatosis, Constrictive Pericarditis

## Background

We have demonstrated that fragmented QRS complexes (fQRS) on a 12-lead ECG correlate with the presence of myocardial scar in coronary artery disease (CAD). However, the extent of fQRS as a marker of non-CAD related myocardial pathology as seen on delayed gadolinium enhancement (GDE) cardiac magnetic resonance imaging (CMR) is unknown.

## Methods

**Figure 1:**
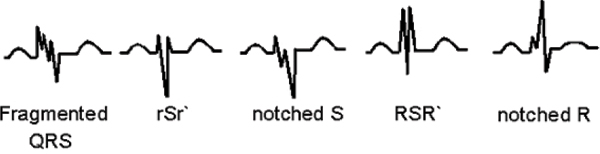
**Different morphologies of fQRS on 12-lead ECG**.

The fQRS on 12-lead ECG was defined as the presence of fragmented QRS, notched R or S wave, or RSR' pattern in at least 2 contiguous leads corresponding to an individual coronary artery region (anterior: V1 to V6 leads, lateral: I, aVL and V5, V6 leads, and inferior: II, III and aVF leads, respectively) in absence of a typical bundle branch block pattern (Figure 1). CMR studies of 91 patients (pts) with GDE (0.1 mmol/kg Gadolinium) and ECG were studied. Presence of non-subendocardial and patchy or diffuse mid to subepicardial GDE patterns was considered abnormal. Pts with CAD, prior myocardial infarction, and transmural/subendocardial GDE were excluded (n = 34).

## Results

Of 57 pts (mean age 41 ± 15 years, 41% male), 17 (30%) pts had fQRS on their 12-lead ECG and 8(14%) pts had abnormal non-CAD-related GDE. The CMR exams were performed for dilated cardiomyopthy (n = 9), myocarditis (n = 5), constrictive pericarditis (n = 6), arrhythmia (n = 14), intracardiac mass (n = 7), sarcoidosis (n = 11), and suspected amyloidosis (n = 2) or hemochromatosis (n = 3). Sensitivity and specificity of detecting non-CAD related myocardial pathology by fQRS on ECG were 88% and 80% respectively *p* < 0.001. Results with analysis of anterior, lateral and inferior fQRS are summarized in Table 1.

**Table 1:** Table 1

	Sensitivity	Specificity	P value
FQRS	88%	80%	<0.001
Anterior fQRS	50%	94%	0.01
Lateral fQRS	67%	98%	<0.001
Inferior fQRS	80%	81%	0.01

## Conclusion

Fragmented QRS complexes on a 12-lead ECG is reliable marker of both CAD and non-CAD related myocardial disease with a high degree of specificity to localize the region of the involved myocardium.

